# Increasing the Construct Validity of Computational Phenotypes of Mental Illness Through Active Inference and Brain Imaging

**DOI:** 10.3390/brainsci14121278

**Published:** 2024-12-19

**Authors:** Roberto Limongi, Alexandra B. Skelton, Lydia H. Tzianas, Angelica M. Silva

**Affiliations:** 1 Department of Psychology, Brandon University, Brandon, MB R7A 6A9, Canada; skeltoab70@brandonu.ca; 2 Department of Psychology, University of Western Ontario, London, ON N6A 3K7, Canada; ltzianas@uwo.ca; 3 Department of French and Francophone Studies, Brandon University, Brandon, MB R7A 6A9, Canada; silvaa@brandonu.ca

**Keywords:** computational phenotypes, computational psychiatry, active inference, computational psychopathology, linguistic phenotypes, free energy principle

## Abstract

After more than 30 years since its inception, the utility of brain imaging for understanding and diagnosing mental illnesses is in doubt, receiving well-grounded criticisms from clinical practitioners. Symptom-based correlational approaches have struggled to provide psychiatry with reliable brain-imaging metrics. However, the emergence of computational psychiatry has paved a new path not only for understanding the psychopathology of mental illness but also to provide practical tools for clinical practice in terms of computational metrics, specifically computational phenotypes. However, these phenotypes still lack sufficient test–retest reliability. In this review, we describe recent works revealing that mind and brain-related computational phenotypes show structural (not random) variation over time, longitudinal changes. Furthermore, we show that these findings suggest that understanding the causes of these changes will improve the construct validity of the phenotypes with an ensuing increase in test–retest reliability. We propose that the active inference framework offers a general-purpose approach for causally understanding these longitudinal changes by incorporating brain imaging as observations within partially observable Markov decision processes.

## 1. Introduction: The Utility of Brain Imaging in Understanding Mental Illness

At the beginning of the last decade of the twentieth century, the field of psychiatry experienced a ‘new wave’ in the understanding and treatment of mental illnesses. This wave took the form of non-invasive and human-centered brain imaging. Basic researchers and clinicians agreed that tools to understand the biological mechanisms underlying the human mind could facilitate evidence-based therapies and biomarkers, transitioning psychiatry from symptom-based definitions to biologically validated constructs of mental disorders [1,2].

The expected utility of brain imaging for understanding mental illnesses has predominantly been statistical. By measuring brain activity, indexed, for example, by blood oxygen level dependent (BOLD) signals (i.e., functional magnetic resonance imaging, fMRI,) during the performance of cognitive tasks, neuroscientists have been able to collect myriads of data points that encode complex associations between brain disfunction, aberrant cognitive performance, and symptoms of mental disorders [3].

At the turn of the century, only the most statistically salient associations were identified using traditional correlational approaches. However, advancements in big-data technologies, such as machine learning, have uncovered distinct features of mental illnesses [4,5,6]. Furthermore, metanalytic works have leveraged 30 years of cumulative data, leading to proposed neural markers shared by several mental disorders [7]. Today, these types of brain activity vs. observed behavior (i.e., brain–symptom) associations continue to prevail in most brain imaging studies of mental illnesses [7,8].

While the scientific utility of brain–symptom correlational approaches is unquestionable, the expectations about their clinical utility have not been met yet [9]. Most setbacks revolve around low specificity, sensitivity, and interrater reliability of diagnosis interpretation [10,11,12]. As an example of the kinds of errors the use of brain imaging can cause in diagnoses, consider the diagnosis of treatment-resistant major depressive disorder. Consistent with the still-prevalent phrenological perspective of functionally localized brain causes of mental illness, aberrant increased activity in the sub-genual anterior cingulate cortex (sgACC) has been reported to be associated with treatment resistance in major depression [7,13]. The cingulate cortex functionally connects with the dorsolateral prefrontal cortex. This implies that by increasing the activity of the prefrontal cortex, the activity of the sgACC would be downregulated, resulting in remission of resistance to (pharmacological) treatment. A recent placebo-controlled study involving repetitive transcranial magnetic stimulation of the dorsolateral prefrontal cortex has shown promising results in this direction [14]. Crucially, in this trial, prefrontal stimulation foci were identified via brain imaging (MRI) at the individual level. Despite this optimistic progress in treatment, the diagnosis of depression based on specific aberrant activity in the sgACC remains either unknown (at best) or unreliable (at worst). At the moment, no consensus has been reached about the specificity and sensitivity rates of aberrant activity of the sgACC in treatment-resistant major depression. Furthermore, aberrant activity in the agACC has been associated with other conditions [15,16,17]. This variability in aberrant cingulate activity among different conditions poses difficulties in the interpretation of imaging data among clinicians (i.e., low interrater reliability). Therefore, relying on the observation of aberrant cingulate activity to diagnose treatment-resistant major depressive disorder could lead to both false negatives and false positives cases. Similar limitations are present in the diagnosis of other disorders (e.g., schizophrenia).

## 2. Computational Psychiatry and Computational Phenotyping

During the last two decades, the emergent field of ‘computational psychiatry’ has opened a new path towards the fulfillment of the expected utility of brain imaging in understanding and treating mental illnesses. Computational psychiatry works speak to two general approaches: theory-driven and data driven. Whereas data-driven approaches follow the steps of machine-learning statistical analysis of brain–symptom associations [18], theory-driven approaches focus on unobserved or hidden variables at the interface between the mind and the brain. A hallmark of theory-driven computational psychiatry speaks to its potential for providing mechanistic neuro-cognitive explanations of the pathophysiology of mental illnesses.

Theory-driven computational psychiatry attempts to explain a wide range of mental disorders (see Table 1 for examples). Theory-driven computational psychiatry targets the identification of hidden or latent variables that explain the mechanistic underpinning of mental illnesses resulting from aberrant behavioral and cognitive states [19,20,21,22,23,24,25,26]. In bipolar disorder, for example, a mood-sensitivity parameter of a dynamical bifurcation model tracks mood oscillation at the individual level [27]. In substance abuse (e.g., alcoholism), reinforcement learning models allow the identification of which behaviors would help a particular patient to abstain from engaging in addictive behaviors [28]. Similarly, in antisocial behavior, deficits in learning from punishment are associated with conduct disorder, and reinforcement learning models allow the identification of the learning speed of a particular individual [29]. In all the above examples, computational psychiatrists tend to refer to a collection of subject-specific and symptom-specific computational parameters as ‘phenotypes’ [30]. For most computational neuroscientists (including the authors of this review), computational phenotyping will become the gold standard for diagnosis and individualized treatments [20,21,31,32]. However, as we discuss below, to reach that point, computational phenotype must increase its construct validity.

## 3. Current Limitations of Computational Phenotyping

Computational psychiatry has progressively paved the way towards clinical trials [22]. However, this path still has challenges to resolve [82]. As in the case of traditional (and machine-learning augmented) statistical brain–symptom approaches, the clinical field of psychiatry continues to expect reliable and valid computational phenotypes (c.f., biomarkers). Most critiques come from the fact that these phenotypes lack test–retest reliability, being particularly unstable (i.e., variable) when estimated at two different times [83,84]. For example, in a recent work, Silva et al. [75] used a Bayes network to assess the dependence between linguistic phenotypes (a set of specific linguistic cues extracted from speech samples produced by patients) and diagnostic accuracy of schizophrenia. They estimated the probability of observing a longitudinal change in specific linguistic cues and the consensus diagnosis after six months from the first psychosis episode. Their work showed a high probability of schizophrenia diagnosis given a longitudinal change in a specific instance of the linguistic phenotype. However, their work did not assess whether the longitudinal change was structural (due to the evolution of the disorder) or was due to random measurement error. Test–retest reliability is particularly important because, even in symptom-based approaches, stable symptoms over time (measured through consistent clinical scores) lead to more reliable diagnoses and treatment decisions [85]. A crucial corollary of this, relevant to the current work, is that if unstable symptoms are observed over time (through repeated measurements), the causes of those changes must be identified.

While we agree with the above limitation, test–retest reliability (and all correlation-based psychometric properties of a test measurement) largely depends on construct validity [86,87,88]. Recent works in computational psychiatry reveal a key limitation of computational phenotypes: their construct validity (i.e., the cognitive mechanism explaining the symptoms) is not yet causally informed by neural validity (i.e., the neural mechanism causing the cognitive dysfunction). In the remainder of this review, we will discuss this limitation and will discuss how the integration of brain imaging into the active inference framework of brain functioning could contribute to improve construct validity trough longitudinal, causal neural validity of computational phenotypes of mental illnesses. To contextualize both discussions, we will first describe a recent work addressing the critique of the test–retest reliability of computational phenotypes in healthy individuals [89] which converges with other independent works with schizophrenia patients. This work unveiled one fact (structural longitudinal changes) that ascribes construct validity a hinging role in achieving test–retest reliability of computational phenotypes of mental illnesses.

### 3.1. Structural Longitudinal Changes in Parameters of Computational Phenotypes in Healthy Subjects and Schizophrenia Patients

In a recent work aiming to assess the test–retest reliability of computational phenotypes in healthy subjects, Schurr et al. [89] fit seven independent computational models to data collected from subjects over three months. The participants performed cognitive tasks on a weekly basis and filled out structured questionnaires about their emotions. The tasks were selected based on their common use in both computational psychiatry and experimental psychology (broadly defined): go/no-go, change detection, random dot motion, lottery ticket, intertemporal choice, two-armed bandit, and numerosity comparison. For each of these tasks, a specific computational model was fitted to the observed behavioral data. For example, whereas the drift diffusion model [90] was used to fit data from the random dot motion task, an uncertainty exploration model [91] was fitted to data from the two-armed bandit task. To assess the test–retest reliability of parameter estimates, Schurr et al. [89] computed intraclass correlations. The results of the test–retest assessments were mixed. Crucially, they found that part of the variability over time was structural, associated with learning and mood effects.

Schurr et al.’s [43] findings are contextualized in healthy subjects, and their methods did not include brain imaging data. In the context of mental illness, specifically schizophrenia, Alonso-Sánchez et al. [92] and Silva et al. [75] reported longitudinal changes in linguistic phenotypes estimated from spoken language productions. Furthermore, Liang et al. [93] and Wang et al. [94] reported longitudinal changes in brain imaging data. The main conclusion reached by these independent works suggests that longitudinal variation in computational phenotypes does not relate to random noise and confirms the importance of studying the structure of the longitudinal changes in computational phenotypes of [84].

### 3.2. Increasing Construct Validity of Computational Phenotypes Through Causal Understanding of Structural Longitudinal Changes

That correlations do not automatically imply causation is a two-century old statistical truth [95,96,97] which in the twenty first century gains especial meaning in the context of computational phenotypes of mental illness. Structural longitudinal changes in computational phenotypes speak to informative dependencies between parameter estimates (c.f., multicollinearity as uninformative dependencies). However, we do not know whether these informative dependencies indicate autocorrelation or causation. Therefore, a necessary step to increase the construct validity of computational phenotypes of mental illness speaks to understanding the causes of their longitudinal changes, if they are present.

Importantly, the fact that longitudinal changes are also observed in functional imaging indicates that computational phenotypes show structural longitudinal changes not only at the level of the mind but also at the level of the brain. This implies that computational phenotypes should include the causes of the longitudinal neural changes. At present, no computational psychiatry work has reported this type of validation, making it, therefore, an imperative future direction in computational psychiatry.

## 4. Future Directions: Causal Understanding of Structural Longitudinal Changes in Computational Phenotypes Through the Integration of Active Inference and Functional Brain Imaging

The most informative dependency for computational psychiatry may be a set of parameters representing the causal relationship between cognitive processes and brain signals [98,99,100], ‘mind–brain causal computational parameters’. At present, most if not all attempts in this direction still show correlational dependencies. Reinforcement learning models represent exemplar cases of this approach [101,102], undoubtedly providing high, but not sufficient, construct validity to relevant computational phenotypes.

One closer step towards the identification of causal computational parameters refers to a recent work on linguistic phenotyping reported by Limongi et al. [32]. By using Bayes network, a causal network, Limongi et al. reported a causal dependency between linguistic phenotypes of conceptual disorganization of schizophrenia patients and parameter estimates of brain connectivity (dynamic causal models of functional magnetic resonance imaging, fMRI). They reported causal parameters between linguistic phenotypes and causal parameters of fMRI. However, their work still lacks the desired mind–brain causal parameters. Moreover, their work is not contextualized in the longitudinal changes in computational phenotypes which, as discussed above, is necessary for increasing their construct validity, and ensuing test–retest reliability. Despite these limitations, Limongi et al. developed their work on the active inference framework of brain functioning [103]. As we elaborate upon below, this framework represents a biologically feasible way to identify computational parameters representing mind–brain causal dependencies in the longitudinal dimension.

The active inference framework of brain functioning [103] regards the brain as a predictive machine equipped with a model of both the external (exteroceptive) and internal (interoceptive) worlds. These models comprise hidden states or beliefs about the causes of sensory (interoceptive and exteroceptive) observations. In active inference, hidden states can be everything the organism has beliefs regarding and some of these can change longitudinally over time, depending on the organism’s actions (behaviors). The organism updates its state beliefs by inferring posterior probabilities of the states, given observations and behaviors. To infer such posterior beliefs over states, the organism minimizes prediction error signals.

From an implementational perspective, belief-updating and longitudinal changes in hidden states occur through variational or marginal message passing [104,105,106] within a partially observable Markov decision process (POMDP) [102,107]. Importantly, active inference may serve as a general-purpose framework applicable to any cognitive process [108]. In principle, any cognitive task otherwise modeled through specific or task-dedicated frameworks can be modeled through active inference (e.g., the drift diffusion model and reinforcement learning models) [102,109,110].

As a biologically plausible generic data analysis procedure, a POMDP allows to assess the causal relationship in the longitudinal dimension by answering what the value of a phenotype parameter would be at time t + 1, given a hypothetical value at time t. A POMDP allows assess to retrospective counterfactuals [96,97,111,112,113] over the structural longitudinal changes in computational phenotypes. We invite the interested reader to explore the implementational technicalities of a POMDP under active inference in dedicated tutorials [102,107].

Furthermore, in active inference, prediction error signals are mathematically linked to postsynaptic membrane potentials and the ensuing firing rates of neurons. This mathematical link allows the establishment, via POMDP, of neural validity to the parameter estimates of computational phenotypes. Moreover, in theory, a POMDP model could be fitted not only to a data set that includes behavioral responses as actions but also to a data set comprising brain images (e.g., BOLD signals). Signals encoded in the brain images would be regarded as observations caused by neuronal activity, collectively regarded as hidden states in the form of brain regions (nodes). From the perspective of an active inference agent housing a generative model of the causes of its observations, brain regions would represent part of the agent’s hidden internal world (states). These states would cause the signals (observations) encoded in the images.

Based on the above theoretical assumptions, a computational modeler could pursue computational fMRI, electroencephalography, or magnetoencephalography (as just exemplar cases) by using the amount of belief-updating in each node (region) of the POMDP as an explanatory variable and use this in a convolution model in the usual way.

## 5. Concluding Remarks: From Computational Psychiatry to Computational Psychopathology

The present review showed key elements to conclude that identifying mind–brain causal parameters accounting for longitudinal variability represents a necessary goal to increase the construct validity of computational phenotypes. Integrating brain imaging with active inference in POMDPs offers a practical pathway to achieving this goal. By pursuing the causal understanding of the longitudinal changes in model parameters relevant to a computational phenotype, the field of computational psychiatry will likely converge with the field of computational psychopathology [114]. While understanding the mechanisms underlying symptoms (psychopathology) is essential, it is insufficient for the diagnosis of a specific mental illness (psychiatry). Given this differentiation, the application of brain imaging in mental illness will continue to be of upmost importance due to its contribution to the mechanistic understanding of the symptoms. This understanding, we believe, will ultimately help the psychiatry field to meet the expected goals that brain imaging brought about in the last decade of the twentieth century.

## Figures and Tables

**Table 1:** Exemplar studies in computational psychiatry aiming to identify computational phenotypes of mental illness.

Psychiatry Disorder	Computational Psychiatry Study
Autism	[4,33,34,35,36,37]
Obsessive–compulsive disorders	[38,39,40]
Anxiety	[41,42,43,44,45,46,47,48,49,50,51]
Abnormal gambling	[52]
Intolerance of uncertainty	[20,53]
Border personality disorder	[54]
Major depression disorder	[55,56,57,58,59,60]
Schizophrenia	[31,32,36,61,62,63,64,65,66,67,68,69,70,71,72,73,74,75,76,77]
Attention-deficit/hyperactivity disorder	[78,79,80]
Motivation disorder	[81]
